# Characterization of a morphogenetic furrow specific Gal4 driver in the developing *Drosophila* eye

**DOI:** 10.1371/journal.pone.0196365

**Published:** 2018-04-27

**Authors:** Ankita Sarkar, Neha Gogia, Kevin Farley, Lydia Payton, Amit Singh

**Affiliations:** 1 Department of Biology, University of Dayton, Dayton, OH, United States of America; 2 Premedical Program, University of Dayton, Dayton, OH, United States of America; 3 Center for Tissue Regeneration and Engineering at Dayton (TREND), University of Dayton, Dayton, OH, United States of America; 4 The Integrative Science and Engineering Center, University of Dayton, Dayton, OH, United States of America; 5 Affiliate Member, Center for Genome Advocacy, Indiana State University, Terre Haute, IN, United States of America; University of Massachusetts Medical School, UNITED STATES

## Abstract

The ability to express a gene of interest in a spatio-temporal manner using Gal4-UAS system has allowed the use of *Drosophila* model to study various biological phenomenon. During *Drosophila* eye development, a synchronous wave of differentiation called Morphogenetic furrow (MF) initiates at the posterior margin resulting in differentiation of retinal neurons. This synchronous differentiation is also observed in the differentiating retina of vertebrates. Since MF is highly dynamic, it can serve as an excellent model to study patterning and differentiation. However, there are not any Gal4 drivers available to observe the gain- of- function or loss- of- function of a gene specifically along the dynamic MF. The *decapentaplegic (dpp)* gene encodes a secreted protein of the transforming growth factor-beta (TGF-beta) superfamily that expresses at the posterior margin and then moves with the MF. However, unlike the MF associated pattern of *dpp* gene expression, the targeted *dpp*-Gal4 driver expression is restricted to the posterior margin of the developing eye disc. We screened GMR lines harboring regulatory regions of *dpp* fused with Gal4 coding region to identify MF specific enhancer of *dpp* using a GFP reporter gene. We employed immuno-histochemical approaches to detect gene expression. The rationale was that GFP reporter expression will correspond to the *dpp* expression domain in the developing eye. We identified two new *dpp-*Gal4 lines, *viz*., *GMR17E04*-Gal4 and *GMR18D08*-Gal4 that carry sequences from first intron region of *dpp* gene. *GMR17E04*-Gal4 drives expression along the MF during development and later in the entire pupal retina whereas *GMR18D08*-Gal4 drives expression of GFP transgene in the entire developing eye disc, which later drives expression only in the ventral half of the pupal retina. Thus, *GMR18D08*-Gal4 will serve as a new reagent for targeting gene expression in the ventral half of the pupal retina. We compared misexpression phenotypes of Wg, a negative regulator of eye development, using *GMR17E04*-Gal4, *GMR18D08*-Gal4 with existing *dpp*-Gal4 driver. The eye phenotypes generated by using our newly identified MF specific driver are not similar to the ones generated by existing *dpp*-Gal4 driver. It suggests that misexpression studies along MF needs revisiting using the new Gal4 drivers generated in our studies.

## Introduction

*Drosophila* eye serves as an excellent model to study patterning, growth, gene expression, function and disease [1–3]. The sequential progression of neuronal differentiation sheds light into the process of neural development and establishment of precise neuronal connections. *Drosophila* eye develops from an eye imaginal disc housed inside the larva [4–7]. It is an excellent example to study sequential differentiation [8, 9], which is also seen in the higher vertebrates [10]. The larval eye imaginal disc undergoes differentiation to form the retinal neurons, which upon pupal metamorphosis develops into the compound eye of the adult fly comprising of around 800 ommatidia [2, 8, 9, 11, 12]. Each ommatidium comprises of approximately 20 cells, which include the photoreceptor neurons, pigment cells, cone cells and bristles [7, 9, 11, 12]. During early third instar, the retinal differentiation in the developing eye imaginal disc initiates as a synchronous wave of differentiation from the posterior margin of the eye disc and refers to as the Morphogenetic Furrow (MF) [2, 11, 12]. This MF moves anteriorly and, results in delineation of retinal fate behind it [8, 9, 11, 13].

Several signaling pathways are involved in MF formation and progression. The MF initiation and progression depends on the expression of *decapentaplegic (dpp)*, which encodes a homologue of secreted Bone Morphogenetic Proteins (BMPs), belongs to the TGFβ superfamily [14]. In the developing eye, *dpp* expression is restricted to a stripe of cells moving along with the MF [11, 15]. In early first instar eye imaginal disc, *dpp* first expresses at the ventral margin [16–18]. In the second instar, prior to initiation of ommatidial differentiation, *dpp* expresses along the posterior and lateral margins of the eye disc [19, 20]. Dpp then activates the expression of *hedgehog (hh)*, a ligand for Hh signaling, and other transcription factors [2, 11–13, 21–24]. Hh, which encodes a secreted protein, plays a critical role in the MF progression. In second instar, *hh* expresses in the peripodial membrane [16], whereas in early third instar eye disc, *hh* expresses in the center of the posterior margin, where it triggers MF formation and its progression [25, 26]. Dpp i-s involved in repression of Wingless (Wg), a ligand for evolutionarily conserved Wg/Wnt signaling pathway, which also works as a negative regulator of MF progression [27–30]. Interestingly, a highly conserved growth regulatory Hippo signaling pathway is also involved in regulation of MF progression. The effector of Hippo signaling pathway, *yorkie (yki)*, can negatively regulate MF progression by activating Wg signaling in the developing eye [31]. In order to study MF, a dynamic morphological landmark, which is yet to be fully understood, there is a need for Gal4 drivers, which can drive expression of transgene along with the MF.

One of the strengths of *Drosophila* model is the availability of large repository of tools like Gal4/UAS system [32–34], which allows study of gain-of-function or loss-of-function of a gene of interest in the domain specific manner in a specific time window of development. The challenge with MF is that it is dynamic, and the available reagents like *dpp*-Gal4 (BL-1553 and others) [35], can mark only one stage of the MF. It drives expression of the transgene only on the posterior margin of the developing eye where the MF is initiated during early third instar of larval eye imaginal disc development[8]. However, in the *dpp*-*lacZ* line, lacZ reporter expression moves with the MF in the developing eye along the temporal axis.

A collection of transgenic lines were generated at the Janelia farm by taking overlapping 3-kb DNA fragments from the flanking noncoding and intronic regions of genes of interest, which were cloned upstream of GAL4, and then inserted into a defined genomic location by site-specific recombination [36]. The rationale was to generate a Gal4 driver line resource to dissect the cis-regulatory modules (CRMs) of the genes of interest and to drive reporter gene expression in a distinct and subset of cells (neuronal populations) with in a developing field [36–38]. Thus, in each line, the expression of GAL4 is under control of a different and defined fragment of genomic DNA, which serves as a transcriptional enhancer [36–38]. We screened these GMR lines for an eye specific enhancer of *dpp*, which will be a great tool for studying regulation of MF formation and progression during patterning and development of the *Drosophila* eye.

Here we present characterization of two eye-specific enhancer lines of *dpp*, *viz*., *GMR17E04*-Gal4 and *GMR18D08*-Gal4. *GMR17E04*-Gal4 drives the transgene expression along the MF and *GMR18D08*-Gal4 drives expression in the entire eye imaginal disc but more robustly on dorso-ventral margins. However, to our surprise, the *GMR17E04*-Gal4 drives expression in the entire pupal retina whereas the *GMR18D08*-Gal4 drives expression only in the ventral half of the pupal retina. *GMR18D08*-Gal4 can also serve as a ventral pupal retinal specific marker. These two CRMs are different from the CRM of *dpp*-*lacZ* reporter, which exhibits eye specific *dpp* expression in *Drosophila*.

## Materials and methods

The stocks used in this study are described in flybase (http://flybase.bio.indiana.edu). The stocks used are UAS-*GFP*-NLS [39], UAS-RFP, *Sp/CyO*; *dpp*-Gal4/TM6B Hu(BL-1553) [35] (a gift from Justin Kumar), *dpp*-*lacZ*/CyO [19, 40], UAS-*wg*-*GFP*[41, 42], UAS-*wg*^RNAi^ [43], UAS-*hpo* [44], and UAS-*yki*^3SA^ [45]. The various GMR CRM lines used are *GMR17E04*-Gal4 (BL-48770), *GMR17G08*-Gal4 (BL-48784), *GMR19B04*-Gal4 (BL-48839), *GMR19D09*-Gal4 (BL-45833), *GMR16G02*-Gal4 (BL-47472), *GMR18B08*-Gal4 (BL-45437), *GMR18D08*-Gal4 (BL-45442), and *GMR19C03*-Gal4 (BL-49283) for *dpp* gene [36]. These *dpp*-CRM lines were generated with the aim to analyze their ability to drive expression of GFP as well as RFP reporter genes. We used an enhancer trap [46] line for *dpp*[17–19]. The flies were maintained on standard fly food at 25°C.

### Genetics

In our studies, we employed a Gal4/UAS system for targeted misexpression [32, 33]. All Gal4/UAS crosses for gain-of-function and loss-of-function were maintained at 18°C, 25°C and 29°C, unless specified, to sample different induction levels [34]. All the targeted misexpression experiments were conducted using the *dpp*-GAL4 driver [35] and other *dpp-*CRM lines [36]. All these Gal-4 lines were crossed individually to UAS-*GFP* [39] line to investigate their expression[34].

### Immunohistochemistry

Imaginal discs were dissected from first-, second-, and third-instar larvae in 1XPBS (Phosphate Buffered Saline) and were fixed in 4% para-formaldehyde for 20 minutes. Imaginal discs were washed in PBS after fixation and stained following the standard protocol [47–49]. Antibodies used were mouse anti-Wg (1:50) (Developmental Studies Hybridoma Bank), mouse anti-Dlg (1:100), mouse anti β-gal (1:100), rabbit anti-Dlg (1:200; a gift from K. Cho), rat anti Elav (1:100). Secondary antibodies (Jackson Laboratories) used in this study were goat anti-rat IgG conjugated with Cy5 (1:250), donkey anti-rabbit IgG conjugated to Cy3 (1:250), donkey anti-mouse IgG conjugated to FITC (1:300), and donkey anti-mouse IgG conjugated to Cy3 (1:200). The imaginal discs were mounted on slides in Vectashield mountant (Vector Laboratories). Immunofluorescent images were obtained using the Olympus Fluoview 1000 Laser Scanning Confocal Microscope[50]. The confocal images were processed using the Photoshop CS6 software.

### Adult eye imaging

Adult *Drosophila* eye images were taken using a Zeiss Apotome Imager.Z1 microscope. The flies were prepared by freezing them at -20°C for around 2 hours. The legs and wings of the flies were removed and flies were mounted on a dissection needle, and the fly was positioned on a glass slide using mounting putty [51–54]. Images were captured by using extended depth of focus function of the Axiovision software version 4.6.3 to generate Z-stacks. The final images and figures were prepared using Adobe Photoshop CS6 software.

## Results

**Fig 1:**
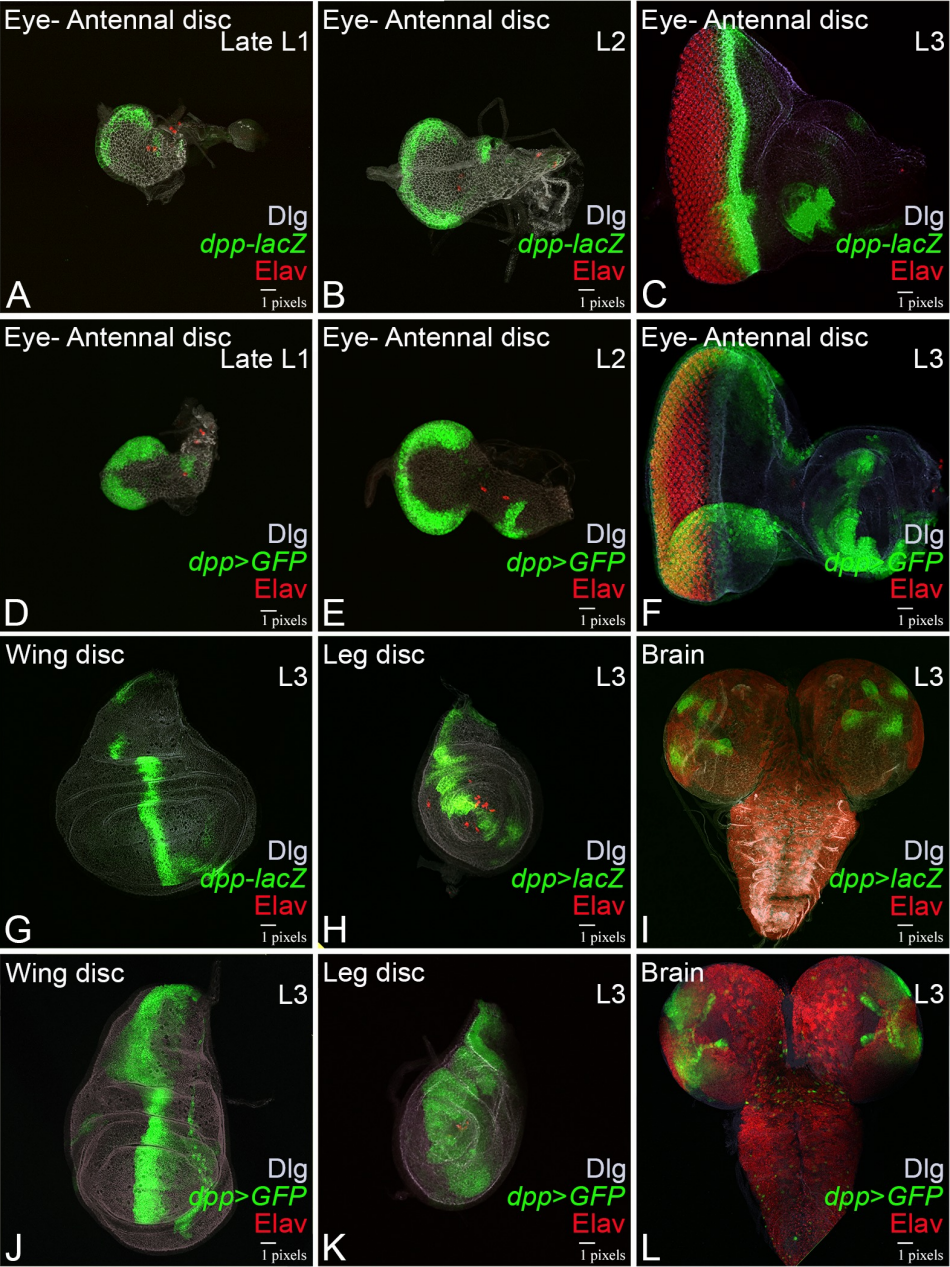
In the developing eye imaginal disc, *dpp*-Gal4 driven GFP reporter does not correspond to *dpp*-lacZ expression. Expression of (A-C) *dpp*-lacZ reporter (Green), (D-F) *dpp*-Gal4 (Green) driver in (A, D) Late first instar- (L1), (B, E) second instar- (L2) and (C, F) third instar- (L3) eye imaginal discs. Note that *dpp*-lacZ expression is initiated at posterior margin of (A) late L1 eye disc, which further evolves and moves with the Morphogenetic furrow (MF) in (B) second instar eye disc and (C) in third instar eye disc. However, *dpp*-Gal4 driver expression is restricted to the posterior margin during all stages of development (D, E, F). A pan neural marker Elav (red) marks the retinal neurons and a membrane specific marker Dlg (white) marks the outline of the disc. In wing imaginal disc both (G) *dpp*-lacZ as well as (J) *dpp*-Gal4 drive GFP reporter (*dpp*>GFP) marks the antero-posterior boundary. In the leg imaginal disc, both (H) *dpp*-lacZ as well as (K) *dpp*-Gal4 drive GFP reporter (*dpp*>GFP) marks the dorsal sector. In larval brain (I) *dpp*-lacZ and (L) *dpp*>GFP is expressed in two lateral and two medial spots in a similar pattern.

We wanted to study the expression of *dpp-lacZ* reporter under the *dpp* enhancer [19, 40] with GFP reporter expression under the *dpp*-Gal4 (*dpp*>*GFP*) driver in the larval brain, eye-, leg- and wing- imaginal disc (Fig 1). The *dpp-lacZ* expression is initiated at the ventral posterior margin of the late first instar (Late L1) eye-antennal imaginal disc (Fig 1A), which further evolves and marks the entire posterior margin of the late second instar (L2) eye-antennal disc (Fig 1B). At this stage, retinal differentiation has not been initiated based on lack of Elav expression, a pan neural marker that marks the retinal neurons in the developing eye disc. The *dpp*-*lacZ* expression evolves and moves along with the MF in the third instar (L3) eye-antennal disc (Fig 1C). The *dpp*-*lacZ* marks the MF, which is present anterior to the larval retinal neurons in the eye marked by Elav. Expression of GFP reporter transgene under *dpp*-Gal4 enhancer (*dpp*>*GFP*) is similar to *dpp*-*lacZ* expression in the late first instar eye-antennal disc (Fig 1D) and late second instar (L2) eye-antennal disc (Fig 1E). Unlike *dpp*-*lacZ*, *dpp*>*GFP* expression fails to move with the MF from the posterior margin of eye imaginal disc to the anterior in the third instar (L3) eye-antennal disc. However, *dpp*>*GFP* expression remains restricted to the posterior margin of the eye disc and does not move along with the MF (Fig 1F). In the larval wing disc, *dpp* is expressed as a narrow stripe in the middle, which marks the antero-posterior (AP) compartmental boundary. Both *dpp*–*lacZ* (Fig 1G) and *dpp>GFP* (Fig 1J) exhibits similar expression along the border of the AP compartmental boundary [19, 40]. In the leg disc, *dpp* is expressed in the dorsal sector[55]. Both *dpp*-*lacZ* (Fig 1H) and *dpp>GFP* (Fig 1K) exhibits similar expression in the dorsal sector/region. In the larval brain, *dpp* is expressed in two lateral and two medial spots, which is similar for both *dpp-lacZ* (Fig 1I) and *dpp>GFP* (Fig 1L). With respect to other tissues *dpp*-*lacZ* expression in wing- (Fig 1G) leg- imaginal disc (Fig 1H) and brain (Fig 1I) is similar to that of *dpp*-Gal4 driven GFP reporter (Fig 1J, 1K and 1L). We also used another reporter RFP (UAS-RFP) to validate our results (data not shown). Thus, even though *dpp*-Gal4 drives *dpp* expression domain in the wing disc, leg disc and larval brain similar to *dpp-lacZ*, it still does not represent the expression of *dpp*-enhancer in the third instar (L3) eye imaginal disc. Therefore, it is not an optimal driver for the MF specific expression.

### Screening GMR lines for *dpp* eye enhancer

We therefore screened the expression of *dpp-*CRM (Cis Regulatory Module) lines from GMR collections (Table 1) [36] for driving expression of UAS-*GFP* transgene [39]. These lines carry different overlapping domains of the upstream region of *dpp*, the gene of interest (Table 1), and are tagged to GAL4 driver [36]. We analyzed expression of these lines by crossing these *GMR* lines with UAS-*GFP*[39] transgenes.

**Table 1:** List of *dpp* CRM lines analysed in this study.

BDSC Stock No.	Symbol	Id	Seq._coord of fragment	Primer 1, Primer 2 (used to make fragment)	Fragment length	Orientation of fragment
48770	P{GMR17E04-GAL4} attP2	GMR17E04	2L:2428913..2432834	gagtggatatccgagtcgaaccagt,ccactctgactaactggaaaatccc	3921	inverted
48784	P{GMR17G08-GAL4} attP2	GMR17G08	2L:2450278..2451074	ggaagcgactcggctgattggatac,actcggaaagttggggctttagccc	796	same
48839	P{GMR19B04-GAL4} attP2	GMR19B04	2L:2432214..2435785	gcaaagcggattgattaggggtcgt,ccctcaaagcgttccgattggatcg	3571	inverted
45833	P{GMR19D09-GAL4} attP2	GMR19D09	2L:2435128..2438996	ctacggccgaaagtggaaaaatctg,ccaacccaatttggcaccttgttaa	3868	same
47472	P{GMR16G02-GAL4} attP2	GMR16G02	2L:2425041..2428154	agctcttccttcggcggtgtctcct,cactgccgaccacgatggcaagttg	3113	same
45437	P{GMR18B08-GAL4} attP2	GMR18B08	2L:2455899..2457734	ccaagtcggccaacacagtgcgaag,gcgggaatgctcttcacgtcgaagt	1835	same
45442	P{GMR18D08-GAL4} attP2	GMR18D08	2L:2446783..2449086	gcataactcgaacgcctcttgccat,cagttcttcacttgtcgccgtctgt	2303	same
49283	P{GMR19C03-GAL4} attP2	GMR19C03	2L:2440970..2444735	ctaccctcgtcctcaccacctatca,gagggattgcgcgtatcagcctcga	3765	same

dpp enhancers lines stocks in Bloomington Drosophila Stock Centre (BDSC)

• FBgn 0000490

• CG9885

We analyzed the GFP reporter gene expression driven by all these Gal4 lines in the developing eye-antennal imaginal disc (S1 Fig), wing imaginal disc (S2 Fig), leg imaginal disc (S3 Fig), haltere imaginal disc (S4 Fig), and third instar larval brain (S5 Fig). We found that among these eight *dpp* CRM transgenic lines in GMR collection (available at Bloomington Stock Center fly collection) (Table 1), only two *GMR18D08*>*GFP* (S1 Fig) and *GMR17E04>GFP* (S1 Fig) exhibit eye specific expression. However, the other *dpp* CRM lines like *GMR18B08*-Gal4 (S1 Fig), *GMR19D09*-Gal4 (S1 Fig), *GMR16G02-*Gal4 (S1 Fig), *GMR17G08-*Gal4 (S1 Fig), *GMR19B04-*Gal4 (S1 Fig) *and GMR19C03-*Gal4 (S1 Fig) did not show any eye specific expression. The *GMR17E04-*Gal4 marks the larval brain (S5 Fig). *GMR18B08-*Gal4 also shows robust expression in the larval brain (S5 Fig). None of these lines exhibit expression of *dpp* in the wing imaginal disc (S2 Fig), leg imaginal disc (S3 Fig) or haltere imaginal disc (S4 Fig). We have further analyzed GFP reporter expression driven by these two *dpp*-Gal4 lines along the spatio- temporal axis.

### Spatio-temporal profiles of new eye specific enhancers of *dpp*

**Fig 2:**
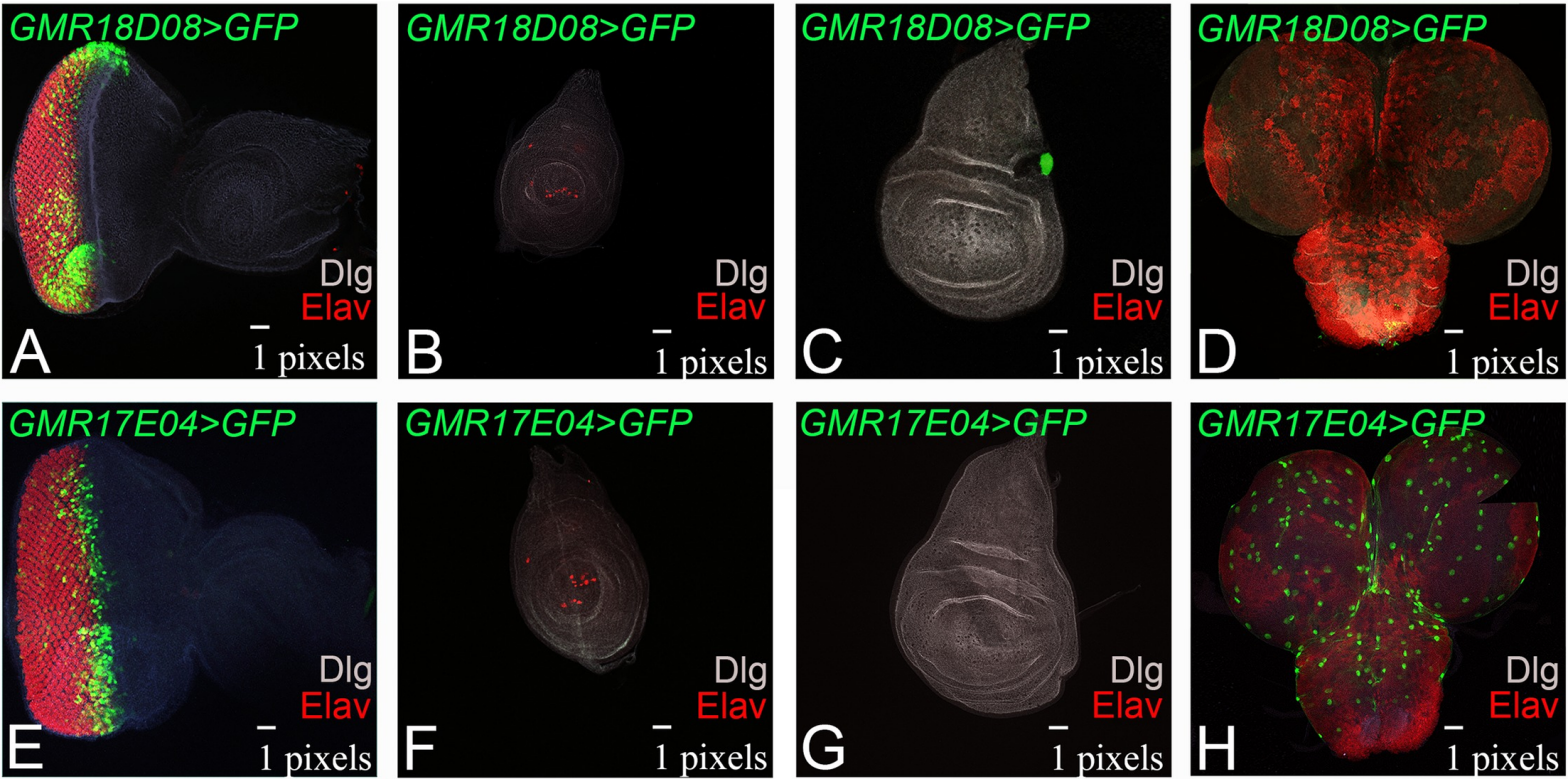
Expression of two eye specific GMR lines in third instar imaginal disc. Expression of (A-D) GMR18D08>GFP, (E-H) GMR17E04>GFP in (A, E) eye imaginal disc, (B, F) leg imaginal disc, (C, G) wing imaginal disc, and (D, H) brain. Note that (A) GMR18D08-Gal4 drives GFP reporter in the entire eye imaginal disc whereas in (E) GMR17E04-Gal4 drives GFP reporter only along morphogenetic furrow (MF). Note that both the GMR lines do not express in leg imaginal disc though GMR18D08-Gal4 shows a patch in the wing disc. However, GMR17E04-Gal4 is also expressed in the brain.

Next, we analyzed expression of these two eye specific enhancer lines like *GMR17E04*-Gal4 and *GMR18D08*-Gal4 in the third larval instar stage. During late second or early third instar, the MF is initiated at the posterior margin of the developing eye-antennal imaginal disc [7–9]. We found that *GMR18D08>GFP* expresses strongly on the dorso-ventral (DV) margin of the eye imaginal disc and in the entire eye disc (Fig 2A). Furthermore, *GMR18D08*-Gal4 does not direct GFP reporter expression in the L3 leg disc (Fig 2B), or larval brain (Fig 2D) and is specific to the developing eye. Although a small dot like region at the border of wing blade along with hinge region exhibit *GMR18D08 >GFP* transgene expression (Fig 2C). In case of *GMR17E04>GFP*, a strong GFP expression was seen along the MF in the developing eye imaginal disc (Fig 2E). The *GMR17E04>GFP* driver that marks MF does not drive GFP expression in the leg (Fig 2F) or wing disc (Fig 2G) but shows GFP expression in the larval brain (Fig 2H). We also verified these expression domains using UAS-FRP transgene (data not shown).

**Fig 3:**
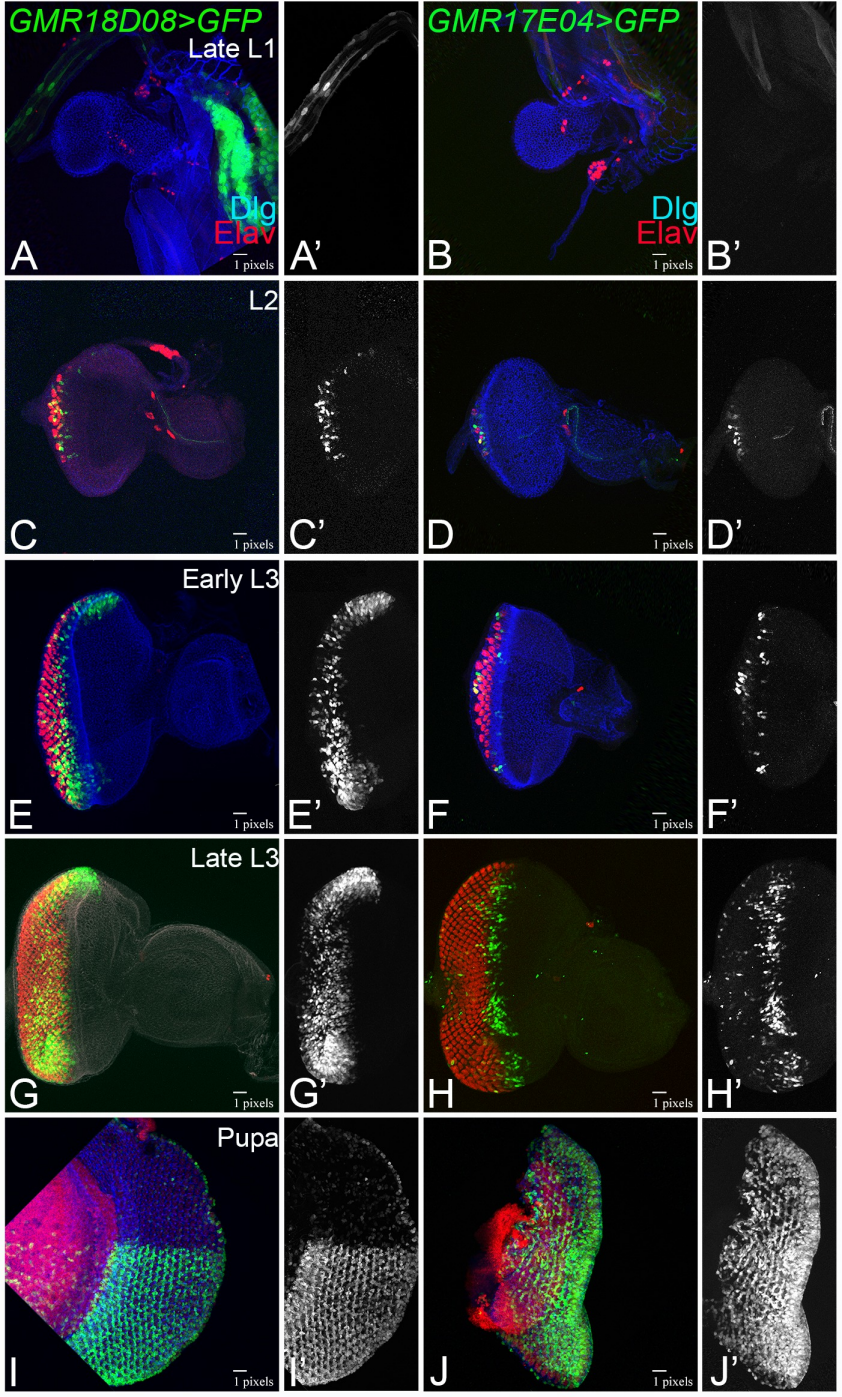
Spatio-temporal profile of eye specific enhancers in the developing eye. GFP reporter expression driven by (A,C,E,G,I) GMR18D08>GFP and (B,D,F,H,J) GMR17E04>GFP in (A,B) late L1, (C, D) L2, (E,F) Early L3, (G,H) late L3 larval eye imaginal disc and (I, J) pupal retina. Note that GMR18D08 does not drive GFP expression in (A) early L1 disc, which then (C) initiates GFP expression on the posterior margin of L2 eye disc, expresses in entire (E) early L3 disc, (G) late L3 eye disc. (I) GMR18D08>GFP expresses in ventral half of the pupal retina. The *dpp* enhancer line GMR17E04>GFP initiate GFP expression in (D) posterior margin of L2, (F) moves with the MF in early L3, (H) Late L3, and (J) expresses in entire pupal retina. The discs were stained for pan neural marker Elav (Red) and membrane specific marker Dlg (Blue).

The two *dpp* enhancers lines—*GMR18D08-*Gal4 (Fig 2A; S1 Fig) and *GMR17E04-*Gal4 (Fig 2E; S1 Fig, Table 1) can regulate GFP reporter expression along the temporal axis in the developing eye imaginal disc. To determine, if these *dpp*-CRM lines have similar expression domains as of *dpp* gene during all stages of eye development, we further extended our analysis during larval eye development. The *GMR18D08>GFP* does not drive GFP expression in the late first instar eye disc (Fig 3A and 3A’). The *GMR18D08>GFP* expression is initiated in a small group of cells along the posterior margin of the second instar developing eye imaginal disc (Fig 3C and 3C’). In early third instar stage, *GMR18D08>GFP* expresses in the entire eye disc behind the MF (Fig 3E and 3E’). In the late third instar, *GMR18D08>GFP* expression is in the entire eye region of the eye imaginal disc (Fig 3G and 3G’). Interestingly, in the pupal retina, *GMR18D08>GFP*, GFP expression is restricted to the ventral half (Fig 3I and 3I’). The other *GMR17E04>GFP*, does not drive GFP reporter expression in the late L1 eye imaginal disc (Fig 3B and 3B’) but its expression is initiated on the posterior margin in the late second instar eye imaginal disc (Fig 3D and 3D’). It evolves in the early third instar eye imaginal disc, where it moves along with the MF (Fig 3F and 3F’) and then in the late third instar *GMR17E04>GFP* marks only the MF (Fig 3H and 3H’). In the pupal stage, the *GMR17E04>GFP* drives GFP expression in the entire pupal retina (Fig 3J and 3J’). Interestingly, *GMR17E04-*Gal4 CRM line driven GFP reporter expression mimics *dpp* expression in the developing eye imaginal disc along the spatio-temporal axis.

### Identification of new eye specific CRM of *dpp*

**Fig 4:**
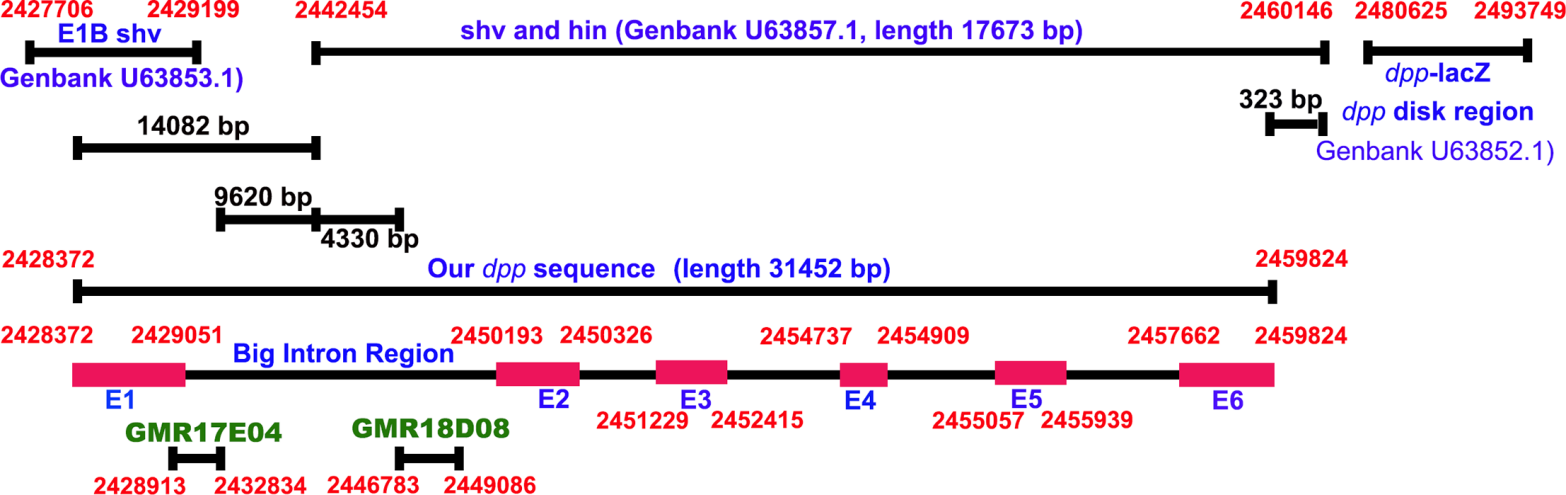
Schematic presentation of *dpp* enhancer and CRM lines using GMR collections. The two newly identified *dpp* CRM lines are *GMR18D08*, *GMR17E04*[56] are in one big intron region in *dpp* gene sequence of *Drosophila melanogaster’s* 2L chromosome. Both of these CRM drive expression in the developing eye. These CRMs do not overlap with each other and are present upstream of known *dpp* CRM element(s) shown in BS 3.0 lacZ (GenBank U63852.1) in 3’ region of disk *dpp*.

The *dpp* gene has been divided into three major regions, *viz*., shortvein (shv); haploinsufficiency (hin); and imaginal disk specific-disk (disk). These two newly identified *dpp* CRMs in the eye, *viz*.,—*GMR17E04*-Gal4 (total size of 3921 base pairs[56]) and *GMR18D08*-Gal4 (total size of 2304 base pairs[56]) (Fig 4, Table 1). These two *dpp* CRM lines are present inside one big first intron of *dpp* gene sequence on 2L chromosome from position 2428913 to 2432834 and 2446783 to 2449086, respectively. Furthermore, *GMR17E04*-Gal4 sequence is present upstream of *GMR18D08*-Gal4 and these two CRMs do not overlap with each other [56](Fig 4). We found that these two CRM lines differ from the existing *dpp*-*lacZ* line, which is 3.0 construct generated from disk region of *dpp* located at 3’ end [19]. This *dpp*-*lacZ* contains a 3’CRE located on 2L chromosome from 2480625 to 2493749 (total size of 13125 base pairs) can drive the *dpp-lacZ* expression specifically along the MF in the eye-antennal disc. We found that the sequence of the two newly identified *dpp* CRMs are located upstream and is different from the known CRM of *dpp*-lacZ in the disk region.

### Targeted expression of Wg with the MF specific marker does not affect the eye fate

**Fig 5:**
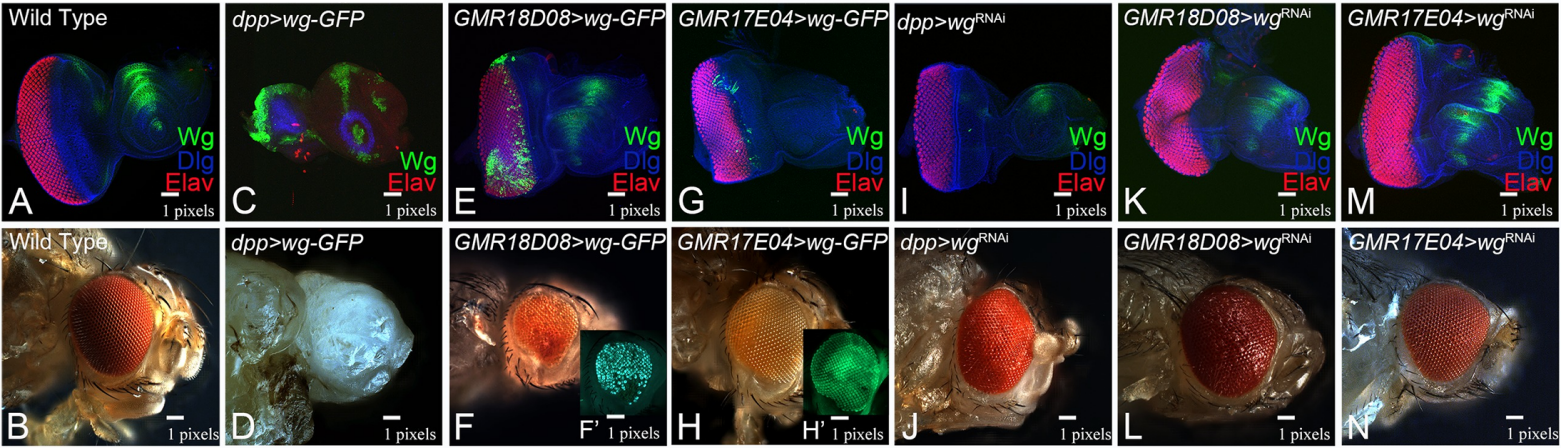
Comparison of phenotypes of gain-of-function and loss-of-function of *wg*, a negative regulator of eye development, using *dpp*-Gal4 with newly identified *dpp-*enhancer Gal4 lines. (A-B) wild-type (A) third instar larval eye imaginal disc stained for Dlg (Blue), Elav (Red). (C-H) Gain-of-function of *wg* using GFP tagged *wg* (UAS -*wg* -GFP) with (C, D) *dpp*-Gal4, (E, F) *GMR18D08-*Gal4, (G, H) *GMR17E04*-Gal4 driver. Note that *dpp*-Gal4 driven GFP (*dpp>wg*-GFP) result in a “no-eye” phenotype as seen in (C) the imaginal disc and (D) the adult eye. The *GMR18D08*>*wg*-GFP results in the (E) near wild-type eye imaginal disc and (F) reduced adult eye with preferential loss of ventral eye. The *GMR17E04*>*wg*-GFP results (G) in slightly reduced eye disc and (H) significantly reduced adult eye due to preferential loss of ventral eye. (I-N) Loss-of-function of *wg* using UAS-*wg*^*RNAi*^ transgene driven by (I, J) *dpp*-Gal4, (K, L) *GMR18D08*-Gal4, (M, N) *GMR17E04*-Gal4 drivers. Note that (I, J) *dpp*>*wg* ^*RNAi*^ results in slight enlarged eye disc and the adult eye, (K, L) *GMR18D08*>*wg* ^*RNAi*^ results in eye field enlargement as seen in (I) the eye disc and (J) the adult eye, (M, N) *GMR17E04*>*wg* ^*RNAi*^ results in subtle enlargement of (M) eye-antennal imaginal disc and the (N) adult eye.

Wg, a negative regulator of eye development, is known to suppress the eye fate upon misexpression in the eye [28, 29]. In comparison to the wild-type eye imaginal disc (Fig 5A) and adult eye (Fig 5B), targeted misexpression of *wg* tagged with GFP in the eye using *dpp*-Gal4 line (*dpp>wg-GFP*) suppresses the eye fate and results in “no- eye” as seen in the eye imaginal disc (Fig 5C) and the adult eye (Fig 5D). Gain-of-function of *wg* using *GMR18D08-Gal4* (*GMR18D08>wg-GFP)*, which marks the ventral half of pupal retina, results in near wild type eye imaginal disc (Fig 5E). However, in the adult eye *GMR18D08>wg-GFP* exhibits reduced eye phenotype due to preferential loss of the ventral eye (Fig 5F). The *wg* transgene is tagged with GFP reporter, which allows us to verify Wg misexpression by *GMR18D08* driver by looking at GFP expression (data not shown). The other *dpp* enhancer, *GMR17E04-*Gal4 driver (*GMR17E04*>*wg-GFP*), which marks MF (does not completely suppress the eye fate in the eye imaginal disc (Fig 5G), significantly affect the eye size in the adult eye (Fig 5H). Loss-of-function of *wg* in the eye using *dpp*-Gal4 driver (*dpp>wg*^*RNAi*^) results in enlargement of the eye imaginal disc (Fig 5I) and the adult eye (Fig 5J). Loss-of-function of *wg in GMR18D08>wg*^RNAi^ resulted in subtle eye enlargement phenotype as seen in the eye imaginal disc (Fig 5K) and the adult eye (Fig 5L). Loss-of-function of *wg* using the *GMR17E04*-Gal4 (*GMR17E04*>*wg*^RNAi^) exhibits wild-type phenotype both in the eye imaginal disc (Fig 5M) and the adult eye (Fig 5N). Our data suggests that negative regulators of eye development, if misexpressed along the MF did not dramatically suppress the eye fate as seen with the *dpp*–Gal4 driver that drives expression along the posterior eye margin during eye development.

### Targeted expression of Hippo and Yorkie with the MF specific marker

**Fig 6:**
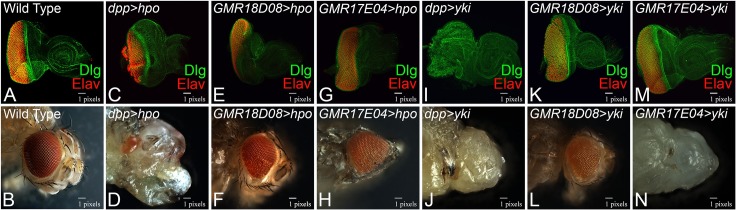
Comparison of phenotypes of gain-of-function of *hpo* and *yki*, which blocks Morphogenetic Furrow (MF) progression, using *dpp*-Gal4 with newly identified *dpp*-enhancer Gal4 lines. (A-B) wild-type (A) third instar larval eye imaginal disc stained for Dlg (Green), Elav (Red). (C-H) Gain-of-function of *hpo* (UAS-hpo) with (C, D) *dpp*-Gal4, (E, F) *GMR18D08*-Gal4, (G, H) *GMR17E04*-Gal4 driver. Note that *dpp*-Gal4 driven *hpo* (*dpp>hpo*) result in a small eye phenotype as seen in (C) the imaginal disc and (D) the adult eye. However, *GMR18D08>hpo* results in the reduced (E) eye imaginal disc and (F) the adult eye with preferential loss of ventral eye. In *GMR17E04>hpo* results in near normal (G) eye disc as well as the (H) adult eye. (I-N) Gain-of-function of *yki* using UAS-*yki* transgene driven by (I, J) *dpp*-Gal4, (K, L) *GMR18D08*-Gal4, (M, N) *GMR17E04*-Gal4 drivers. (I, J) *dpp>yki* results in no-eye phenotype as seen in the eye disc and the adult eye. Note that *dpp>yki* exhibits overgrowth with lack of retinal differentiation. (K, L) *GMR18D08>yki* results in subtle enlargement of (K) the eye disc and (L) the adult eye. Note that enlargement is prominent in the ventral half of the adult eye. (M, N) *GMR17E04> yki* results in eye field as seen in the (M) eye-antennal imaginal disc and the (N) adult eye. Note that *GMR17E04> yki* adult flies fail to eclose and do not have any eye field.

We wanted to verify these results using other genes, by targeting expression of the Hippo signaling pathway member *hippo* (*hpo*), which will trigger cell death and *yorkie (yki*), which is known to suppress the MF progression [31, 57–59] using these two new *dpp* CRM lines. In comparison to the wild-type eye imaginal disc (Fig 6A) and adult eye (Fig 6B), targeted expression of *hpo* in the eye using *dpp*-Gal4 line (*dpp>hpo*) suppresses the eye fate and results in reduced eye as seen in the eye imaginal disc (Fig 6C) whereas the adult fail to eclose and exhibits highly reduced eye (Fig 6D). Gain-of-function of *hpo* using the *GMR18D08-Gal4 (GMR18D08*>*hpo*), which marks the ventral domain of pupal retina, strongly suppress the eye fate in the eye imaginal disc (Fig 6E), and exhibits reduced adult eye due to defects in the ventral eye (Fig 6F). The *dpp*-CRM line that marks MF, *GMR17E04*>*hpo*, does not dramatically affect the eye size as seen in the eye disc (Fig 6G) and the adult eye (Fig 6H). Gain-of-function of *yki* in the eye using *dpp*-Gal4 driver results in the enlargement of eye field due to overgrowth along with lack of retinal differentiation as seen in the eye imaginal disc (Fig 6I) and the adult eye (Fig 6J). Gain-of-function of *yki* using *GMR18D08*-Gal4 (*GMR18D08*>*yki*) exhibits normal eye disc (Fig 6K) but the adult eye exhibits an elongated ventral half (Fig 6L). Gain-of-function of *yki in GMR17E04>yki* had no effect in the eye imaginal disc (Fig 6M) but the adult flies failed to emerge from the white pupa (Fig 6N). Our data suggests that negative regulators of eye development, or the genes that block MF progression, if misexpressed along the MF does not completely block the eye fate as seen with the *dpp* enhancer driving expression along the posterior eye margin during the eye development.

## Discussion

The strength of *Drosophila* as a genetically tractable model depends on the array of genetic tools available for gain-of-function and loss-of-function for a gene of interest along the spatio-temporal axis[1, 3]. The developing *Drosophila* eye has a morphological landmark *viz*., MF, which is an outcome of synchronous differentiation of retinal neurons and serves as an excellent model to study patterning and differentiation [7–9, 11]. However, lack of reagents to target gain-of-function or loss-of-function (by targeting RNAi) along the MF makes it difficult to study patterning and differentiation. The movement of MF depends on positive forces provided by *dpp* and *hh* and the movement is restricted by Wingless (Wg), which is expressed along the antero-lateral region of the eye disc [4, 24, 28, 29]. During eye development, both *wg* and *hh* do not express in the MF exclusively. However, *dpp* exhibits expression along MF. Thus, a MF specific enhancer of *dpp* may serve as an ideal candidate to drive expression along MF. However, the available *dpp*-Gal4 reagent does not drive expression along with the MF (Fig 1).

The *Drosophila* model allow conversion of an old lacZ enhancer trap line [46] with a P-Gal4 driver[60]. We initially attempted conversion of our tested and available *dpp*-*lacZ* insertion line to a *dpp*-Gal4 driver. The rationale was to develop a tool to target expression along MF. However, our attempts towards the conversion of a lacZ enhancer trap line to Gal4 line using targeted transposition did not succeed. It is known that P [Gal-4] -element mobility is significantly lower than that of the p-lacW (lacZ) construct[33, 60]. The frequency of P element conversion is highly dependent on target and donor P-element location. We found that the conversion of *dpp*-*lacZ* to *dpp-*Gal4 is difficult [33, 60].

### New ventral eye specific Gal4 driver

Our studies led to the identification of two new eye specific *dpp* CRMs in the developing eye imaginal disc. One of them *GMR18D08*-Gal4 drives expression in the developing eye imaginal disc. Interestingly, in the pupal retina its expression gets restricted to only the ventral half (Fig 3I and 3I’). We referred this line as ventral eye specific Gal4. To date, only dorsal eye specific Gal4 drivers are available [61]. However, there is no ventral eye specific Gal4 available. We also tested the efficacy of this ventral eye specific Gal4 by driving expression of *wg*, a negative regulator of eye development. We found that *GMR18D08*>*wg* results in a reduced eye phenotype in the adult fly (Fig 5E and 5F). Interestingly, this reduced eye phenotype was restricted only to the ventral half of the adult eye, which corresponds to its domain of expression in the pupal retina. We further verified our reagents using gain-of-function of *hpo* and *yki* (Fig 6). Thus, our studies demonstrate identification of a new ventral eye specific Gal4 line, which can be used for domain specific targeted misexpression studies in the pupal retina and the adult eye. Interestingly, this *GMR18D08*-Gal4 line is highly specific for the pupal retina. Therefore, *GMR18D08*-Gal4 drives domain specific expression of a transgene and can serve as an excellent tool to study neurodegeneration, retinal degeneration and patterning where interestingly the dorsal half of the same pupal retina can serve as the control. It is known that Wg, a ligand for highly conserved Wnt/Wg signaling pathway, negatively regulates MF progression in the developing eye [28, 29, 62]. Thus, gain of function of *wg* in the MF can block its movement and prevent retinal differentiation behind it. The other new CRM identified in our studies, *GMR17E04*-Gal4 can drive expression of GFP reporter along with the MF. Interestingly, misexpression of *wg*, using this *GMR17E04-*Gal4 (*GMR17E04*>*wg*) significantly reduced the adult eye due to loss of ventral half of the eye (Fig 5H). However, *GMR18D08>wg*, a ventral pupal retina specific driver, resulted in reduced eye phenotype due to preferential loss of the ventral eye (Fig 5F). It is possible that gain-of-function of Wg affects the ventral half of the eye more than dorsal. It is possible because Wg regulation in the ventral eye is different from its regulation in the dorsal eye. In the ventral eye, Wg is in a positive feedback loop with Homothorax (Hth), another negative regulator of eye, whereas in the dorsal eye Wg is independent of Hth [63–65]. Therefore, activation of Wg in the ventral may be different from the dorsal half of the eye.

It can explain the reason behind ventral half of the eye being more susceptible to wg gain-of-function using these newly identified CRM lines. To test if this ventral specific gain-of-function phenotype is exclusive to Wg, we tested the phenotypes of gain-of-function of cell death genes *reaper (rpr)[66–68].* The apoptosis causing genes *grim*, *rpr* and *hid* are involved in tissue homeostasis. Activation of *rpr* triggers cell death. We found that misexpression of *rpr* by GMR17E04-Gal4 (MF specific marker) exhibits a normal adult eye (S6E and S6F Fig) whereas the GMR18D08-Gal4, which is ventral pupal retina specific driver, affects the ventral half of the eye (S6C and S6D Fig). Thus, the MF specific driver phenotype is dramatically different from the one seen with the commonly used *dpp*-Gal4 (BL-1553). This *dpp*-Gal4 drives expression of a transgene only on the posterior margin of the developing eye imaginal disc. In case of *wg*, *dpp>wg* results in “no-eye” phenotype as seen in the eye imaginal disc and the adult eye (Fig 5C and 5D). It is possible that continuous misexpression of *wg*, a negative regulator, on the posterior margin of the developing eye imaginal disc might prevent the MF to progress forward and thereby result in “no-eye” phenotype. Therefore, it may be important to revisit the MF specific targeted expression studies using our newly identified Gal4 driver.

Interestingly, these two new eye specific CRM of *dpp* are present in the first big intron, which is far away from the known 3.0 *dpp*-*lacZ* insertion, which is lying in 3’ end near the disk region of *dpp* gene (Fig 4). Thus, these two *GMR18D08*, *GMR17E04* are new eye specific CRM of *dpp* gene. This unravels the complex regulation of *dpp* gene expression, which plays multiple roles during development. It is known that complex regulation of large genes typically contain multiple CRMs[69]. In many cases, a single CRM is responsible for driving expression in specific subsets of cell populations. Furthermore, the CRMs of the same gene may have overlapping spatial and temporal activities and these partially redundant CRMs plays an important role in tightly regulating gene expression patterns [70–72]. Our analysis provides two new tools to study eye development. The Gal4 lines and CRMs identified and analyzed here will provide valuable tools for future experiments along MF as well as the ventral half of the pupal retina. These stocks will allow us to ask questions that are more mechanistic in terms of how each of this pattern forms in the developing eye.

## Supporting information

S1 Fig Study of expression pattern of GMR lines carrying enhancer of *dpp* using GFP reporter in the developing eye imaginal disc. These GMR enhancer lines are carrying CRE sequences of *dpp*- gene (Table 1). Expression of (A) GMR18B08>GFP, (B) GMR18D08>GFP, (C) GMR19D09>GFP, (D) GMR16G02>GFP, (E) GMR17E04>GFP, (F) GMR17G08>GFP, (G) GMR19B04>GFP, (H) GMR19C03>GFP in (A-H) eye imaginal disc. These discs were stained for Wg (Red) and pan neural marker Elav (Blue). Of these, only GMR17E04 exhibits expression similar to *dpp*-lacZ along the MF.
(TIF)

S2 Fig Study of expression pattern of GMR lines carrying enhancer of *dpp* using GFP reporter in the developing wing imaginal disc. Expression of (A) GMR18B08>GFP, (B) GMR18D08>GFP, (C) GMR19D09>GFP, (D) GMR16G02>GFP, (E) GMR17E04>GFP, (F) GMR17G08>GFP, (G) GMR19B04>GFP, (H) GMR19C03>GFP in (A-H) wing imaginal disc. None of these lines exhibit GFP reporter expression in (A-H) wing imaginal disc.
(TIF)

S3 Fig Study of expression pattern of GMR lines carrying enhancer of *dpp* using GFP reporter in the developing leg imaginal disc. Expression of (A) GMR18B08>GFP, (B) GMR18D08>GFP, (C) GMR19D09>GFP, (D) GMR16G02>GFP, (E) GMR17E04>GFP, (F) GMR17G08>GFP, (G) GMR19B04>GFP, (H) GMR19C03>GFP in (A-H) leg imaginal disc. None of these lines exhibit GFP reporter expression in (A-H) leg imaginal disc.
(TIF)

S4 Fig Study of expression pattern of GMR lines carrying enhancer of *dpp* using GFP reporter in the developing haltere imaginal disc. Expression of (A) GMR18B08>GFP, (B) GMR18D08>GFP, (C) GMR19D09>GFP, (D) GMR16G02>GFP, (E) GMR17E04>GFP, (F) GMR17G08>GFP, (G) GMR19B04>GFP, (H) GMR19C03>GFP in (A-H) haltere imaginal disc. None of these lines exhibit GFP reporter expression in (A-H) haltere imaginal disc.
(TIF)

S5 Fig Study of expression pattern of GMR lines carrying enhancer of *dpp* using GFP reporter in the developing third instar larval brain. Expression of (A) GMR18B08>GFP, (B) GMR18D08>GFP, (C) GMR19D09>GFP, (D) GMR16G02>GFP, (E) GMR17E04>GFP, (F) GMR17G08>GFP, (G) GMR19B04>GFP, (H) GMR19C03>GFP in (A-H) larval brain. Only GMR18B08 and GMR17E04 exhibits robust expression in the larval brain.
(TIF)

S6 Fig Gain-of-function phenotype of *reaper (rpr)* using *dpp*-CRM lines exhibits ventral eye loss. (A, B)*dpp>rpr*, (C,D) *GMR18D08>rpr*, (E,F) *GMR17E04>rpr*. Note that *dpp>rpr* results in highly reduced eye as seen in (A) the eye imaginal disc and (B) the adult eye. (C, D) *GMR18D08>rpr* results in the reduced (C) eye imaginal disc and (D) the adult eye with preferential loss of ventral eye. In *GMR17E04>rpr* results in near normal (E) eye disc as well as the (F) adult eye.
(TIF)
