# Supplementary figures and images for: Characterization of a morphogenetic furrow specific Gal4 driver in the developing Drosophila eye
Source: PLoS One. 2018 Apr 27;13(4):e0196365. doi: 10.1371/journal.pone.0196365 (PMC5922546; doi:10.1371/journal.pone.0196365)

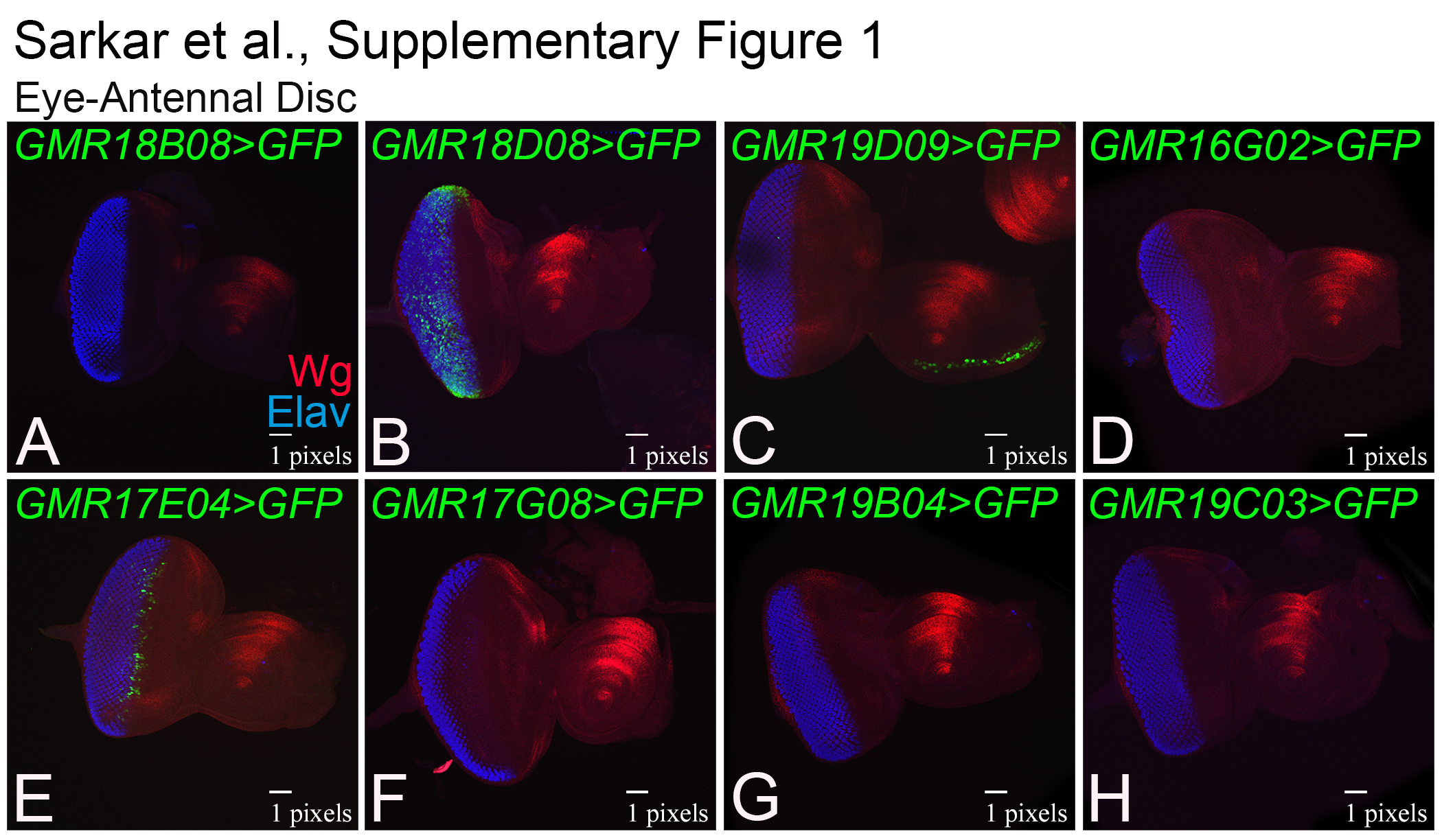

Supplement: S1 Fig — These GMR enhancer lines are carrying CRE sequences of dpp- gene (Table 1). Expression of (A) GMR18B08>GFP, (B) GMR18D08>GFP, (C) GMR19D09>GFP, (D) GMR16G02>GFP, (E) GMR17E04>GFP, (F) GMR17G08>GFP, (G) GMR19B04>GFP, (H) GMR19C03>GFP in (A-H) eye imaginal disc. These discs were stained for Wg (Red) and pan neural marker Elav (Blue). Of these, only GMR17E04 exhibits expression similar to dpp-lacZ along the MF. (TIF) [file pone.0196365.s001.tif]

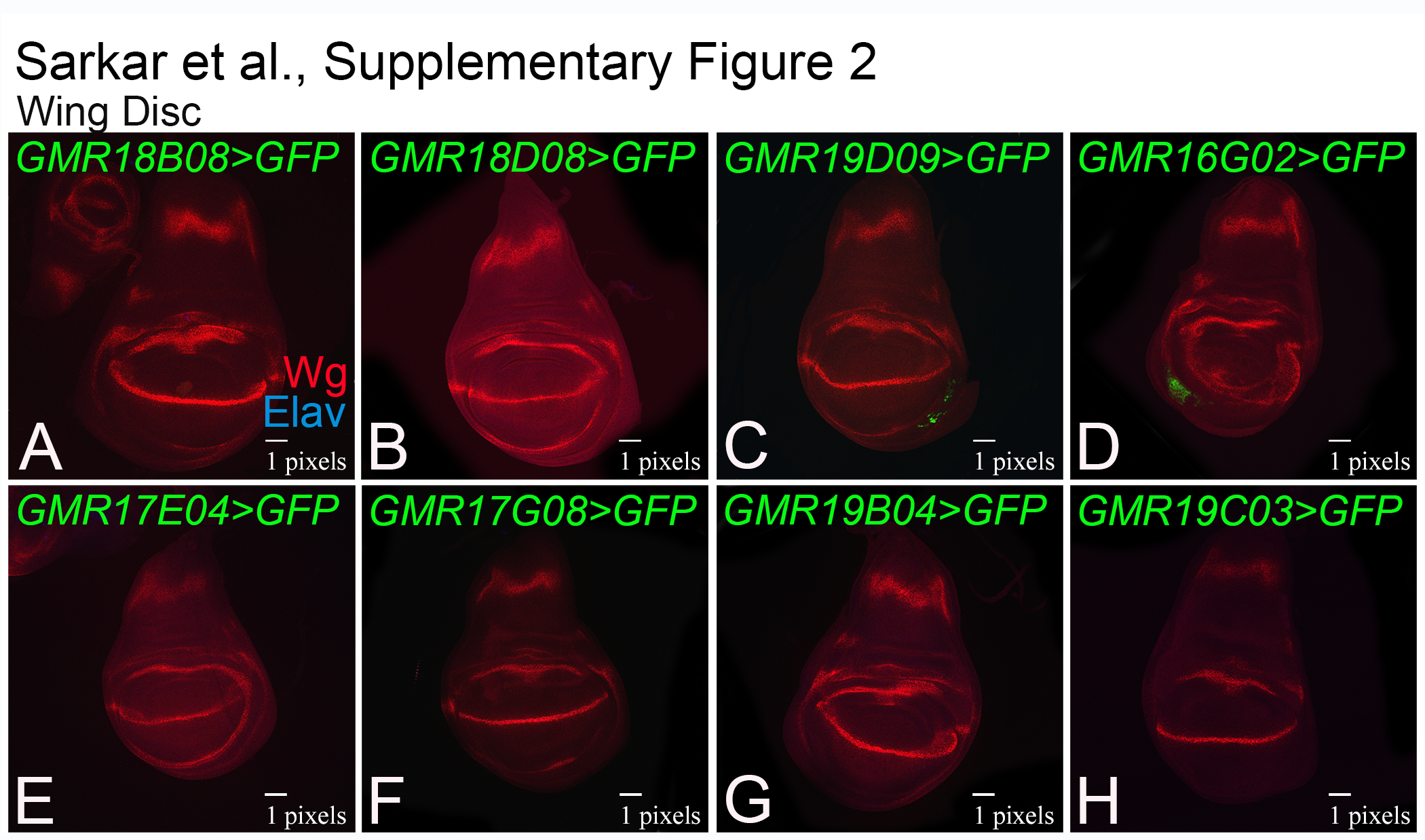

Supplement: S2 Fig — Expression of (A) GMR18B08>GFP, (B) GMR18D08>GFP, (C) GMR19D09>GFP, (D) GMR16G02>GFP, (E) GMR17E04>GFP, (F) GMR17G08>GFP, (G) GMR19B04>GFP, (H) GMR19C03>GFP in (A-H) wing imaginal disc. None of these lines exhibit GFP reporter expression in (A-H) wing imaginal disc. (TIF) [file pone.0196365.s002.tif]

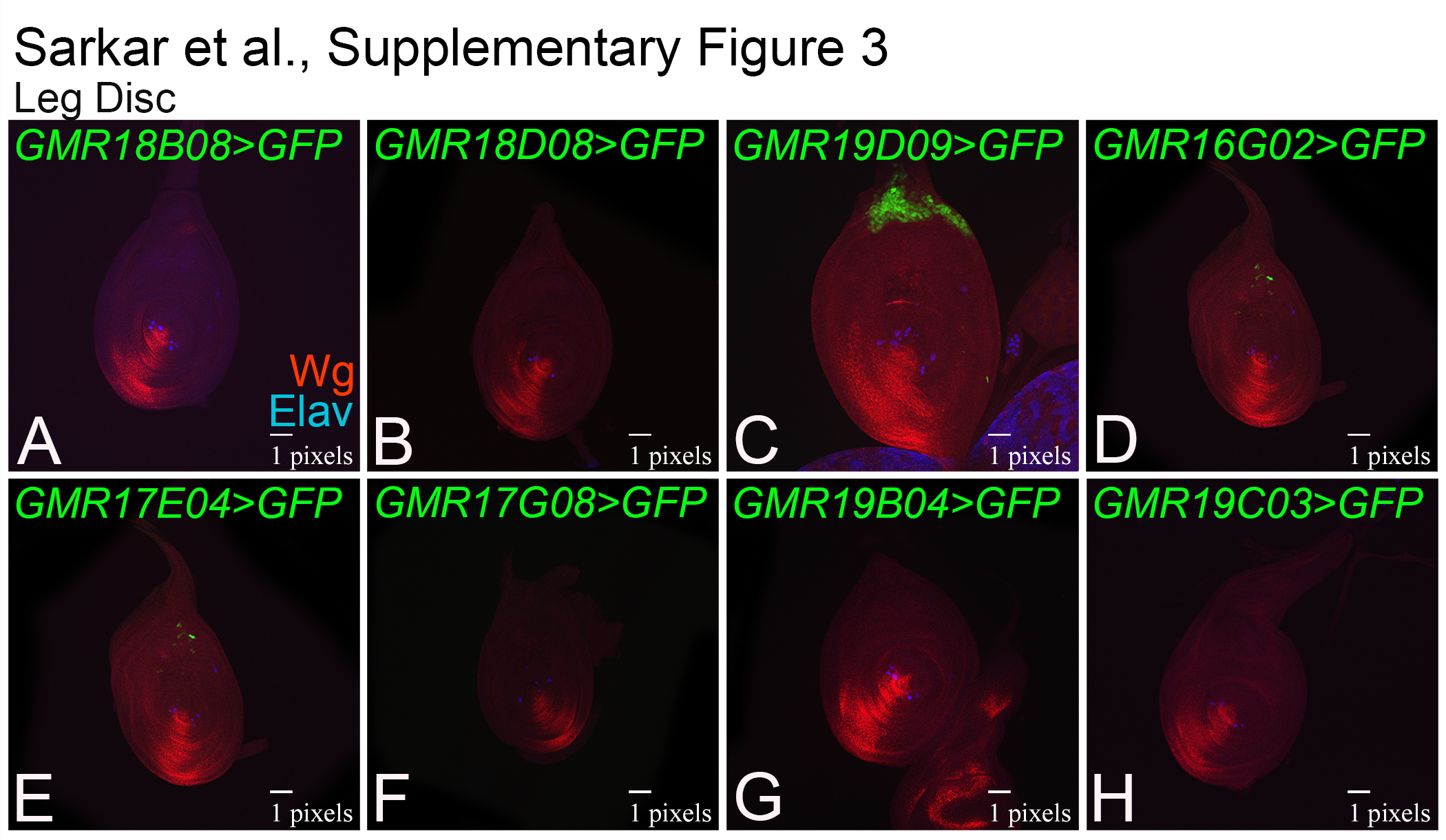

Supplement: S3 Fig — Expression of (A) GMR18B08>GFP, (B) GMR18D08>GFP, (C) GMR19D09>GFP, (D) GMR16G02>GFP, (E) GMR17E04>GFP, (F) GMR17G08>GFP, (G) GMR19B04>GFP, (H) GMR19C03>GFP in (A-H) leg imaginal disc. None of these lines exhibit GFP reporter expression in (A-H) leg imaginal disc. (TIF) [file pone.0196365.s003.tif]

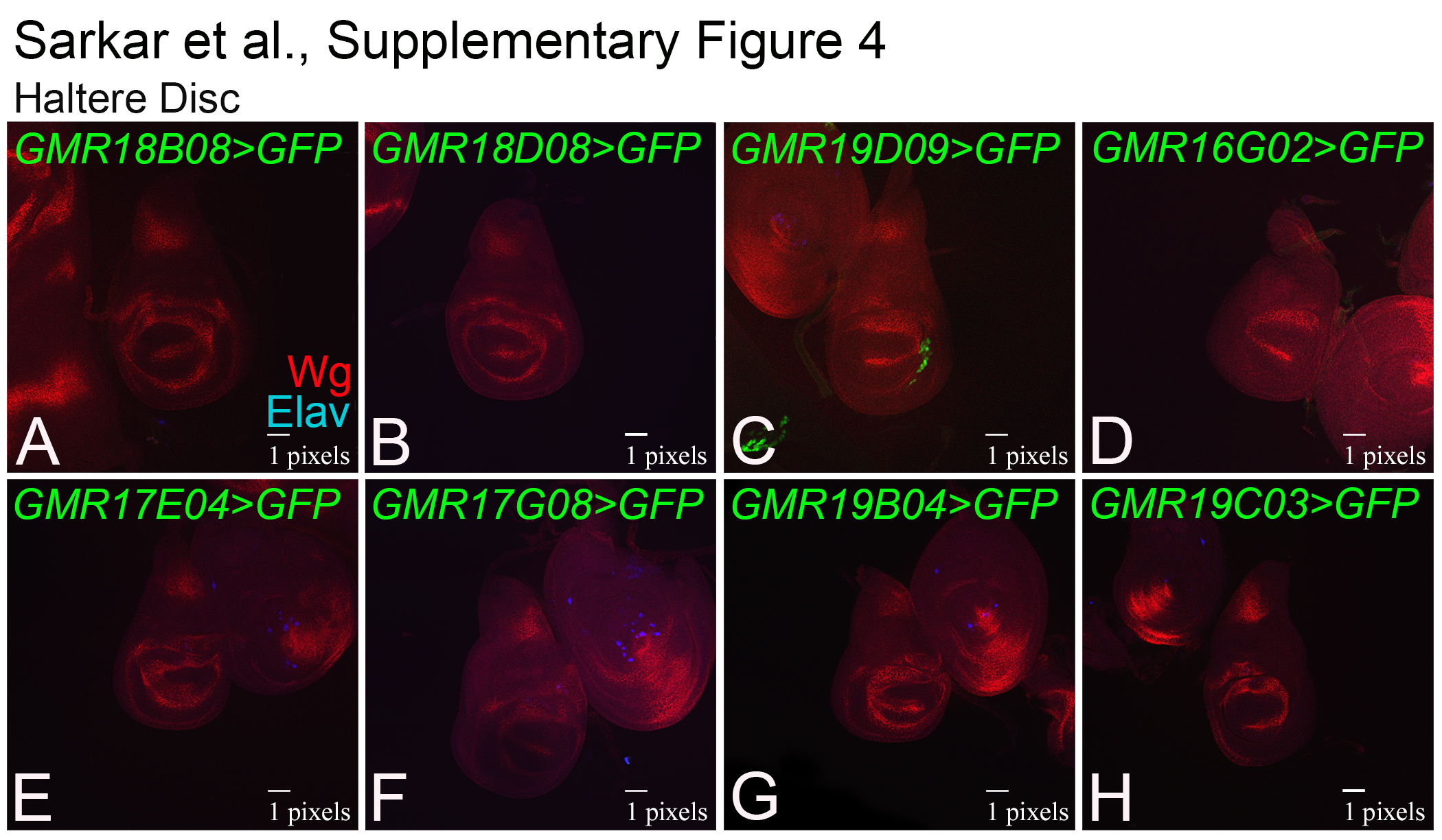

Supplement: S4 Fig — Expression of (A) GMR18B08>GFP, (B) GMR18D08>GFP, (C) GMR19D09>GFP, (D) GMR16G02>GFP, (E) GMR17E04>GFP, (F) GMR17G08>GFP, (G) GMR19B04>GFP, (H) GMR19C03>GFP in (A-H) haltere imaginal disc. None of these lines exhibit GFP reporter expression in (A-H) haltere imaginal disc. (TIF) [file pone.0196365.s004.tif]

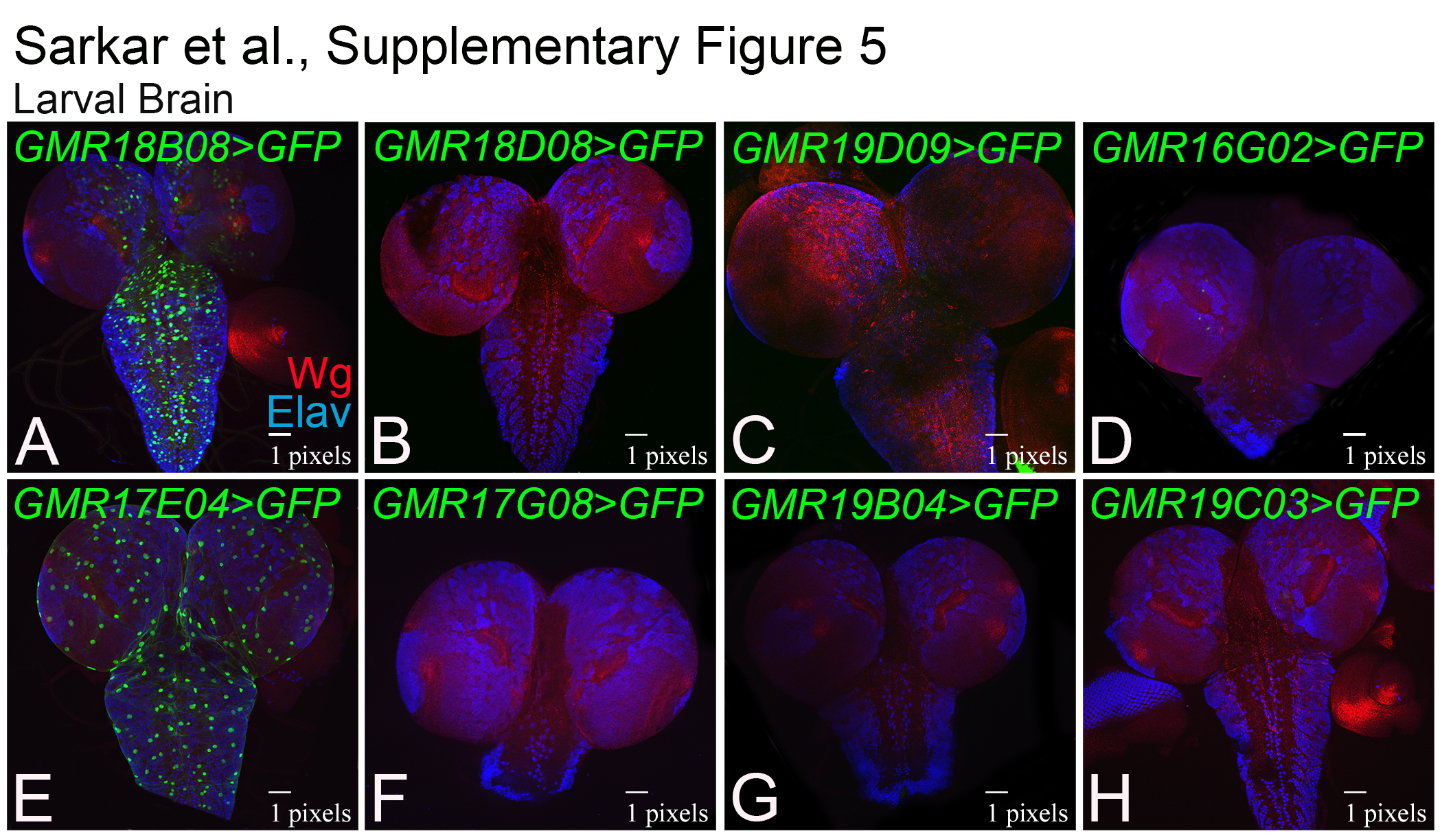

Supplement: S5 Fig — Expression of (A) GMR18B08>GFP, (B) GMR18D08>GFP, (C) GMR19D09>GFP, (D) GMR16G02>GFP, (E) GMR17E04>GFP, (F) GMR17G08>GFP, (G) GMR19B04>GFP, (H) GMR19C03>GFP in (A-H) larval brain. Only GMR18B08 and GMR17E04 exhibits robust expression in the larval brain. (TIF) [file pone.0196365.s005.tif]

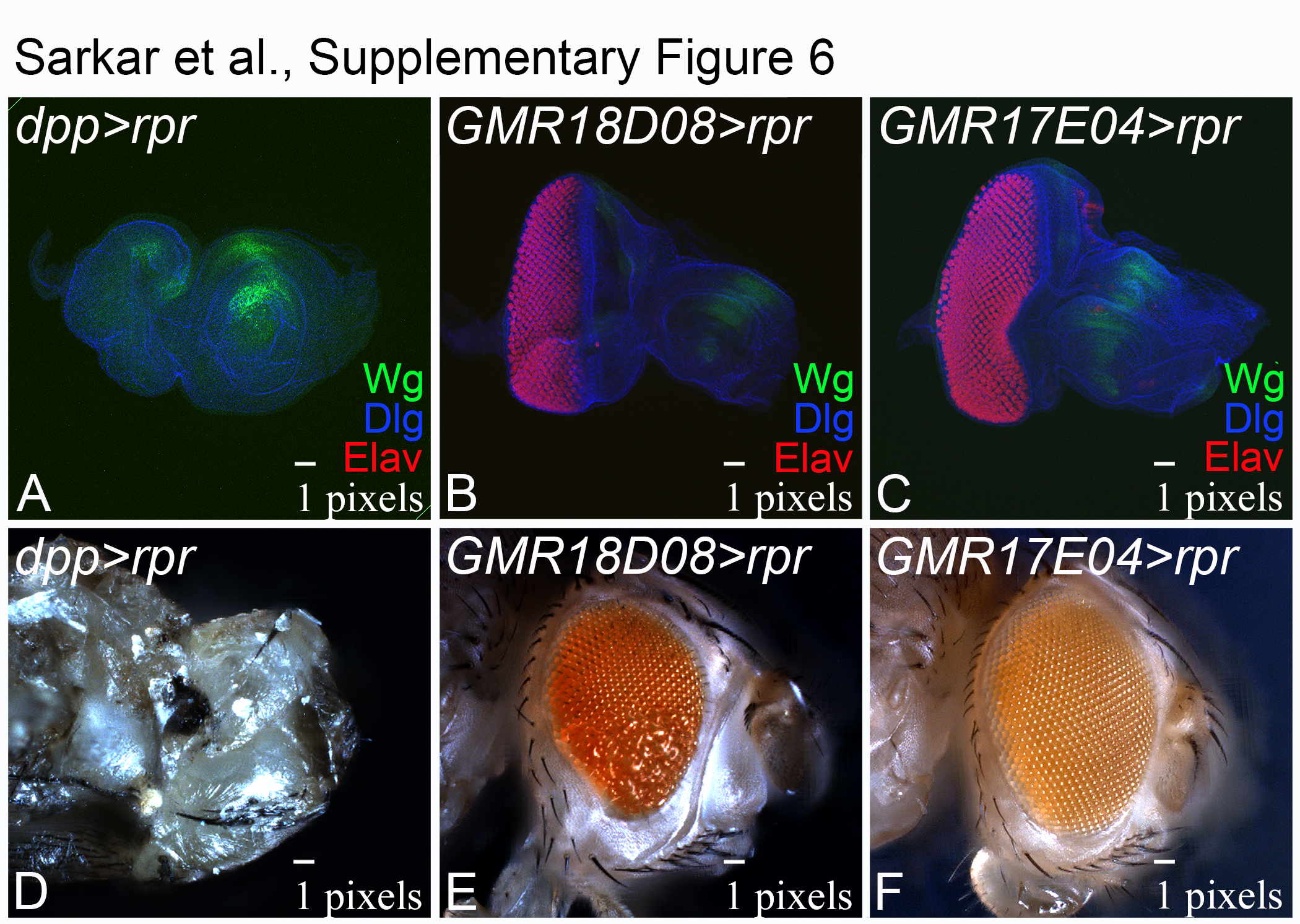

Supplement: S6 Fig — (A, B)dpp>rpr, (C,D) GMR18D08>rpr, (E,F) GMR17E04>rpr. Note that dpp>rpr results in highly reduced eye as seen in (A) the eye imaginal disc and (B) the adult eye. (C, D) GMR18D08>rpr results in the reduced (C) eye imaginal disc and (D) the adult eye with preferential loss of ventral eye. In GMR17E04>rpr results in near normal (E) eye disc as well as the (F) adult eye. (TIF) [file pone.0196365.s006.tif]
